# Mosaicing of Hyperspectral Images: The Application of a Spectrograph Imaging Device

**DOI:** 10.3390/s120810228

**Published:** 2012-07-30

**Authors:** Monica Moroni, Carlo Dacquino, Antonio Cenedese

**Affiliations:** 1 DICEA—Sapienza University of Rome, via Eudossiana 18, Rome 00184, Italy; E-Mail: antonio.cenedese@uniroma1.it; 2 ISPRA—Istituto Superiore per la Protezione e la Ricerca Ambientale, via V. Brancati 48, Rome 00144, Italy; E-Mail: carlo.dacquino@isprambiente.it

**Keywords:** hyperspectral imagery, mosaicing, environmental monitoring, spectrometers, classification

## Abstract

Hyperspectral monitoring of large areas (more than 10 km^2^) can be achieved via the use of a system employing spectrometers and CMOS cameras. A robust and efficient algorithm for automatically combining multiple, overlapping images of a scene to form a single composition (*i.e.*, for the estimation of the point-to-point mapping between views), which uses only the information contained within the images themselves is described here. The algorithm, together with the 2D fast Fourier transform, provides an estimate of the displacement between pairs of images by accounting for rotations and changes of scale. The resulting mosaic was successively georeferenced within the WGS-84 geographic coordinate system. This paper also addresses how this information can be transferred to a push broom type spectral imaging device to build the hyperspectral cube of the area prior to land classification. The performances of the algorithm were evaluated using sample images and image sequences acquired during a proximal sensing field campaign conducted in San Teodoro (Olbia-Tempio—Sardinia). The hyperspectral cube closely corresponds to the mosaic. Mapping allows for the identification of objects within the image and agrees well with ground-truth measurements.

## 1. Introduction

Proximal and remote sensing refer to the science of obtaining information concerning an object, area, or phenomenon through the analysis of data acquired by a device that is not in contact with the investigated subject [1]. In particular, proximal sensing has many interesting small-(laboratory) and medium-(field survey) scale applications. Through the analysis of high spectral resolution data, spectrum characteristics can be identified that allow for the quantitative evaluation of biochemical and biophysical variables related to the physiological state of soils and vegetation. The development of hyperspectral sensors with high spatial and spectral resolution has also enabled the implementation of field investigations for the identification of species, mapping of vegetation cover, forest monitoring, and understanding biogeochemical cycles and their relationships with other sectors of the biosphere [2]. Monitoring and management of natural disasters, *i.e.*, to detect forest fires for suppression and damage mitigation, is another field of application of proximal and remote sensing of great interest currently [3].

In this study, we present an effective methodology for land classification and monitoring that is particularly suitable for investigating large and extensive areas (more than 10 km^2^). This methodology is based on the use of an innovative experimental device for acquiring and analyzing hyperspectral images. The device employs two ImSpector spectrometers; one spectrometer is centered in the visible range of the electromagnetic spectrum (400–1,000 nm), and the other device is centered in the near-infrared region (900–1,800 nm). Each imaging spectrograph is combined with an appropriate monochrome matrix camera equipped with an objective lens to form a spectral imaging device. This system simultaneously acquires the spectra of a line [4]. Multiple images must be acquired to reconstruct a two-dimensional scene based on the combination of several lines. To this aim, a synchronized high resolution camera equipped with a standard lens moving in unison complements the device.

The system constructed from commercially available components has several unique and qualifying characteristics:

- low cost compared to other systems available on the market;
- high spectral resolution;
- high spatial and temporal resolution;
- portability, *i.e.*, the system has been engineered to be transported on ultra-light airplanes.

For aircraft measurements, the instrument is usually mounted on vibration dampening mounts and geolocation of collected data is derived with data collected from a Global Positioning System (GPS) mounted on the same base plate as the camera [5]. The aircraft altitude and pointing information are measured at a frequency lower or equal to the camera frame rate. The architecture of a multi-sensing system used in conjunction with a light aircraft is described in [6]. The navigation data are obtained by an integrated GPS/IMU (Inertial Measurement Unit) that locates the aircraft position and keeps track of the airplane's tilt. To avoid the use of a GPS system and to match the spectral and geolocation data acquisition frequency, the instrument presented in this paper employs the camera equipped with a standard lens and synchronized with the spectral devices for georeferencing.

**Figure 1.**
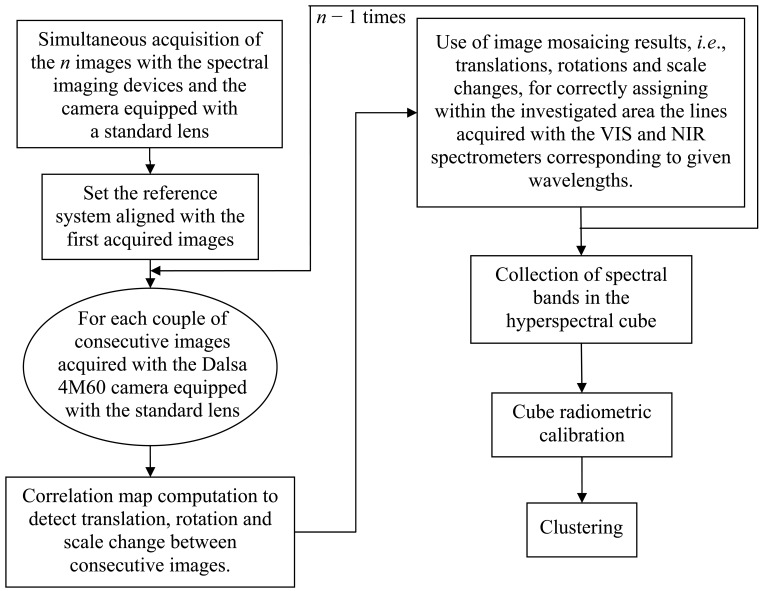
Schematic diagram explaining the main steps in the algorithm.

This paper describes the procedure for combining information contained in multiple, overlapping images of the same scene to produce a single image representing the entire investigated area (*i.e.*, frame fusion) and how this information can be transferred to a push broom type spectral imaging device to build the hyperspectral cube of the area. Figure 1 presents a schematic diagram explaining the main steps in the algorithm.

Two forms of frame fusion are reported in the literature: image mosaicing and super-resolution [7]. The former refers to the alignment of multiple images into a large composition that represents part of a scene. The latter method restores poor-quality video sequences by modeling and removing the degradation inherent to the imaging process. This restoration is achieved by incremental spatial sampling of scene portions and the combination of information from multiple images.

The mosaicing method employed and described here involves images that can be registered using planar homography [8]. A robust and efficient algorithm for image mosaicing, written in the Matlab programming environment and based on the use of the 2-D Fast Fourier Transform (2-DFFT), was designed to automatically register multiple images using only the information contained within the images themselves (no ground control points are required). Acquired images that present a common portion may be rotated and/or translated. Furthermore, the size of the objects present in multiple images is not necessarily conserved. The correlation map provided by 2-DFFT indirectly allows for the evaluation of translations, rotations and scale changes between images. Image mosaicing results, *i.e.*, translations, rotations and scale changes between couples of consecutive images, were finally used for correctly assigning the line acquired with the spectrometer within the investigated area. The application of Fast Fourier Transform for frame fusion techniques, in particular within super-resolution algorithms to reconstruct high resolution images from a series of low resolution images is presented in [9]. To the authors knowledge, FFT is used here for the first time to automatically mosaic thousands of images and georeference spectrometer data.

The accuracy of this method is carefully investigated using portions of a sample image that are transformed using rotation and a change of scale to test the ability of the algorithm to detect the correct extent of superposition.

This paper is organized as follows: Section 2 describes the hyperspectral device with two spectrometers. Section 3 presents an outline of image mosaicing issues and describes the novel algorithm. Sections 4 and 5 describe the algorithm performances evaluated using a sample image and image sequences acquired during a proximal sensing field campaign conducted in San Teodoro (Olbia-Tempio—Sardinia), respectively. Possible algorithm improvements are discussed in the concluding section.

## 2. Hyperspectral Device with Two Spectrometers

**Figure 2.**
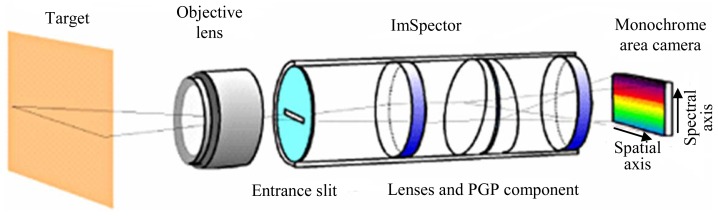
Sketch of a spectrometer [4].

The hyperspectral system is based on the use of two spectrometers (Figure 2); the first spectrometer (VIS) is centered in the visible range of the electromagnetic spectrum (400 nm to 1,000 nm), and the second spectrometer (NIR) is centered in the near infrared region (from 900 nm to 1,800 nm). Each spectrometer captures a line image of a target and disperses the light from each line image pixel into a spectrum. Each spectral image contains then line pixels in a spatial axis and spectral pixels in a spectral axis. A 2D spectral image sequence can be formed by sequentially acquiring images of a moving target or by moving the push broom spectral device.

**Figure 3.**
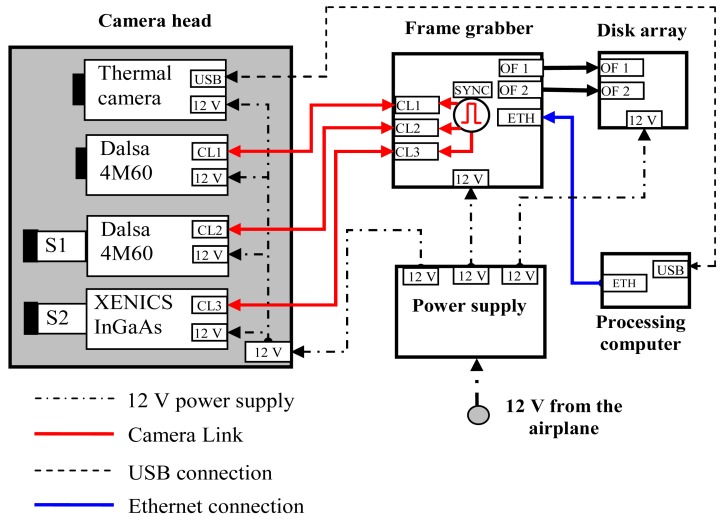
Diagram of the hyperspectral device with two spectrometers. CL stands for Camera Link, OF: optical fiber, SYNC: synchronization signal, ETH: Ethernet plug.

Figure 3 shows a diagram of the system configuration:

- one VIS spectrometer (S1) mounted in front of a Dalsa 4M60 CMOS camera (2,352 × 1,728 pixels @ 25 fps, spectral resolution up to 3 nm);
- one NIR spectrometer (S2) mounted in front of a Xeva Xenics InGaAS camera (640 × 512 pixels @ 25 fps, spectral resolution up to 3 nm);
- one Dalsa 4M60 CMOS camera (2,352 × 1,728 pixels @ 25 fps), equipped with a standard lens, for image mosaicing and georeferencing of the lines acquired by the spectrometers;
- one high-speed DVR (frame grabber) with three Camera Link inputs (IO Industries DVR Express^®^ Blade) used to trigger camera acquisition and to manage the data acquired by the cameras;
- one 1-terabyte solid state disk array;
- one thermal camera;
- one power supply for all system devices;
- one processing computer for controlling the entire system and acquiring images from the thermal camera via a USB port (the thermal images are not discussed in this contribution).

The spectral devices and camera equipped with standard lens simultaneously acquire images at a rate of 25 frames per second. The synchronization signal is generated by the frame grabber. The disk array is suitable for storing up to three hours of acquisitions.

## 3. The Image Mosaicing Algorithm

Three basic steps are required to construct a mosaic: registration, reprojection and blending. The objective of the registration step is to place every image into a global coordinate frame that contains the entire scene. This process requires that an accurate point-to-point correspondence is found between images within the input sequence. The correspondence problem can be stated as follows: given two different views of the same scene, for each image point in one view, find the image point in the second view that corresponds to the same actual point in the scene [7]. In general, such mapping is complex because the correspondence should be defined for all image points. After registration, every point in each image must be transformed (reprojected) to a point in the global frame. The final stage is to blend the images together in their overlapping portions.

The entire procedure allows transforming image pixel coordinates from the old (**P** = (x,y)^T^) to the new (**P**′ = (x′,y′)^T^) reference system [10]. Some of the most common global transformations are related by a homography (similarity, affine, projective) or by polynomial functions [11]. To determine the parameters which characterize these transformations, the correspondence between a certain number of points has to be defined. Polynomial transformations were not considered because they require a larger number of matching points, are more unstable, require more intensive calculations, and provide the least acceptable results relative to the projective transformation.

According to [7], image-to-image mapping is captured by a planar homography when a plane is viewed under an arbitrary camera motion or an arbitrary 3D scene is viewed by a camera rotating about its optical center and/or is zooming. A third situation in which a homography may be appropriate is when a freely moving camera is imaging a distant scene, such as in aerial or satellite photography. The distance between the scene and the camera would be much greater than the motion of the camera between views, assuming that parallax effects caused by the three-dimensionality of the scene are negligible.

The main assumption in this approach is that images are acquired by a perspective pin-hole camera; *i.e.*, the lines connecting all object points and their corresponding images intersect at the camera center. Real cameras may deviate significantly from this pin-hole model due to radial distortion at the periphery of the image. This deviation determines the incorrectness in the mapping views using a homography. This risk may be eliminated by employing high-quality lenses.

Homographic transformation methods that can be employed are as follows [12]:

- Similarity (roto-translation with a change of scale): at least two points are needed to compute four unknown parameters;
- Affine (roto-translation with a change of scale and shear effects): at least three points are needed to compute six unknown parameters;
- Projective (similar to the affine transformation but with a finite point of view): at least four points are needed to compute eight unknown parameters (homography with 8 degrees of freedom).

Corresponding points can be detected via feature-based methods that estimate sparse image features, points and lines. In some cases, these points can be automatically detected using a Harris corner detector ([13,14] or through edge alignment and correspondence-based approaches [15,16]. An estimation method based on a direct comparison between the current original image and the previously calculated mosaic was also proposed [17,18]. The analytical minimization criterion was designed to optimize the determination of the blending coefficient. The challenge in implementing these algorithms is to obtain an accurate and reliable detection of image features and produce a robust and efficient matching of corresponding features in two or more views. The application of this procedure may be difficult when the mosaicing procedure is applied to thousands of images.

Alternatively, the photometric consistency between the image pair provides the parameters for the proper transformation. This approach is suggested when a large number of images were collected. The software developed computes image correlation by means of 2-DFFT. A key element of the method is the assumption of an uncalibrated camera. No prior knowledge of the camera parameters, its motion, optics or photometric characteristics is required. The discrete counterpart of 2-DFFT, the two-dimensional discrete Fourier transform (2-DDFT), is widely used for analyzing 2D signals, including images. 2-DDFT is the series expansion of an image function (over the 2D space domain) in terms of “cosine” image (orthonormal) basis functions. Along with the complex result, the amplitude, phase and power of the transformed data may be computed and compared to detect the image transformations.

The mosaic is obtained through a two-step procedure. First, the reciprocal positions of subsequent images of the acquired sequence, which usually consists of more than 1,500 images, is determined via the 2-DFFT for detecting the maximum spatial correlation between image pairs. To account for eventual rotations around the camera axis and variations in the distance between the point of view and the scene, one image of the pair is rotated in the range of −5° to 5° with a 0.05° step, and the scale is modified relative to the original size of −6% to 6% with a step of 0.5%. The 2-DFFT procedure is iteratively applied to each image pair to select the image pair that maximizes the consistency of the luminosity. As a matter of fact, this is equivalent to a four parameter homographic transformation (similarity). The global frame was chosen to be axis-aligned with the first of the input images. The implemented image blending function used a simple averaging of intensity values. In fact, the algorithm was built essentially to provide information to be used to assign the line acquired with the spectrometers. Mosaic rendering is not problematic provided the mosaic is clear enough to localize the area under investigation. On the other hand, blending plays a major role when images acquired by sensors sensitive within different spectral bands have to be fused [19].

For the second step, consisting of geo-referencing of the mosaic and the hyperspectral cube within the WGS-84 geographic coordinate system datum, ground control points are user selected using a georeferenced map of the area (e.g., a map obtained from Google Earth). In this case, the images are related by eight degrees of freedom for the homography. The result is transformed into a .kmz format to facilitate its visualization.

It is worth noting that being the correlation signal computed from discrete data, the displacement peak (maximum of the correlation signal) is localized on a discrete mesh which leads to an uncertainty of ±0.5 pixel on the displacement measurement. While sub-pixel Gaussian estimators, typically employed in Particle Image Velocimetry algorithms [20], may provide an accuracy of the order of 1/10th of a pixel, they were not implemented so far since the map obtained from Google Earth has a spatial resolution lower than the mosaic. A further release of the software will include a sub-pixel estimator to refine the displacement computation.

## 4. Tests on Sample Images

**Figure 4.**
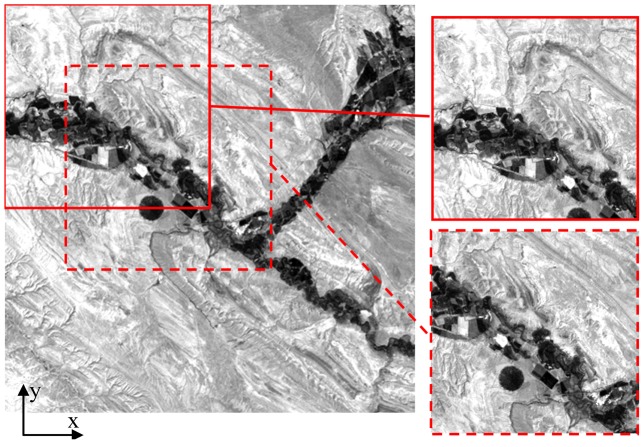
512 × 512 pixel image used for testing of the mosaicing algorithm; an example is shown of a pair of sub-images employed for the algorithm test.

The algorithm was tested using the sample image shown in Figure 4. The goal of this test was to investigate the algorithm performance with image pairs cropped from the sample image with varied extents of overlap (from 50% to 100%). The effects of translation, rotation and change of scale of the second sub-image with respect to the first sub-image have been considered. For each test, the correlation peak magnitude and its shift from the map center have been stored to perform the comparisons.

Two 256 × 256 pixel sub-images have been cropped from the original image. The first sub-image comprises the upper-left part of the image. The second portion is

- translated with respect to the first sub-image from zero to a maximum of 100 pixels in the x, y and oblique (45°) directions;
- rotated by up to 5° in both clockwise and counterclockwise direction with a 0.1° step relative to the first one;
- scaled within the interval (−6% to 6%) with a 1% step.

Two pairs of images that are translated by 75 pixels in the 45° clockwise direction are outlined in Figure 4 using solid and dotted lines. Combinations of movements were also considered.

**Figure 5.**
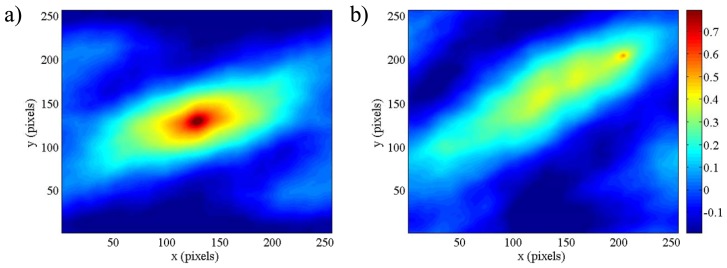
Cross-correlation map for a pair of images with (**a**) 100% and (**b**) 50% overlap.

Figure 5 shows the cross-correlation map for the pair of images with 100% (left figure panel) and approximately 50% (right panel) overlap. The color bar is the same for both figures. The general behavior of the correlation function in the case of translation along the x axis of the second image with respect to the first one is characterized by a maximum value of the correlation peak for the null translation or a maximum corresponding to the displacement of the second image in the translation direction. Figure 5(a) shows that the highest peak (where one represents the result of the cross-correlation of the same image) is located in the center of the image (*i.e.*, where no translation occurs for the pair).

Conversely, Figure 5(b) presents a smoother peak located in the position denoting a translation of 75 pixels in the x direction and 75 pixels in the y direction between the pair of images. Both results are consistent with the imposed displacements (refer to Figure 4 for the x-y reference system orientation).

**Figure 6.**
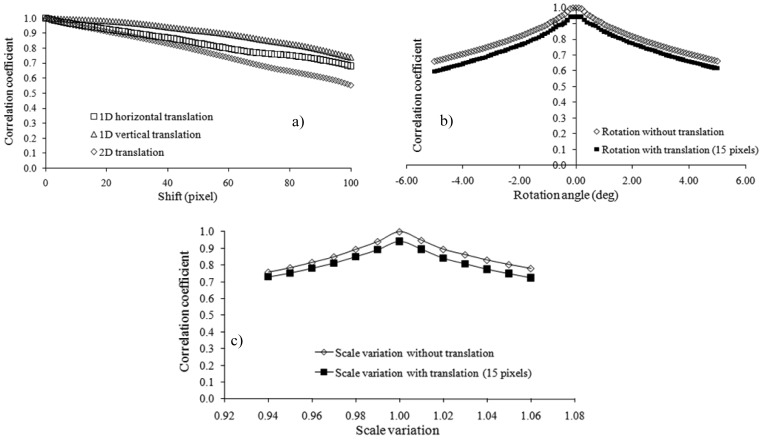
Effects of the extent of overlap between image pairs on the correlation coefficients.

Figure 6 synthesizes the results obtained for different translations, rotations and changes of scale. Figure 6(a) presents the behavior of the correlation coefficient as a function of the image shift in the x, y and 45° directions. As expected, the correlation coefficient decreases with a linearly increasing shift between the image pairs. This finding is due to the reduced degree of overlap between the images. The decrease in the correlation coefficient is less consistent for translations in the y direction. The differential behavior of the correlation coefficient in various directions is dependent only on the sample image employed for the tests.

Figure 6(b) describes the dependence of the correlation coefficient on the rotation angles. Only the rotation is considered in one case, whereas in the other case, rotation is combined with image translation. The general behavior of the correlation function in the case of the rotation of the second image with respect to the first one is characterized by larger correlation peak values for null rotation and null translation. For small angles (between −0.2° and 0.2°), the correlation coefficient presents a maximum that is equal to one in the case of rotation without translation. The measure of the similarity between images shows a steep decrease with an increasing rotation angle. If images are also translated (15 pixels in the x direction in this example), the correlation coefficient decreases for all rotation angles while maintaining a maximum value for the rotation angle set to zero. The lack of symmetry for clockwise and counterclockwise rotations again depends on the sample image.

Figure 6(c) reports the dependence of the correlation coefficient on scale variations. Again, translation increments the image inconsistency. The matching measure is maximized for no changes of scale both with and without translation. Small-scale variations result in decreases in the correlation coefficient. The results are affected by the presence of translation (in this case, 15 pixels in the x direction) over-imposed onto the change of scale.

**Figure 7.**
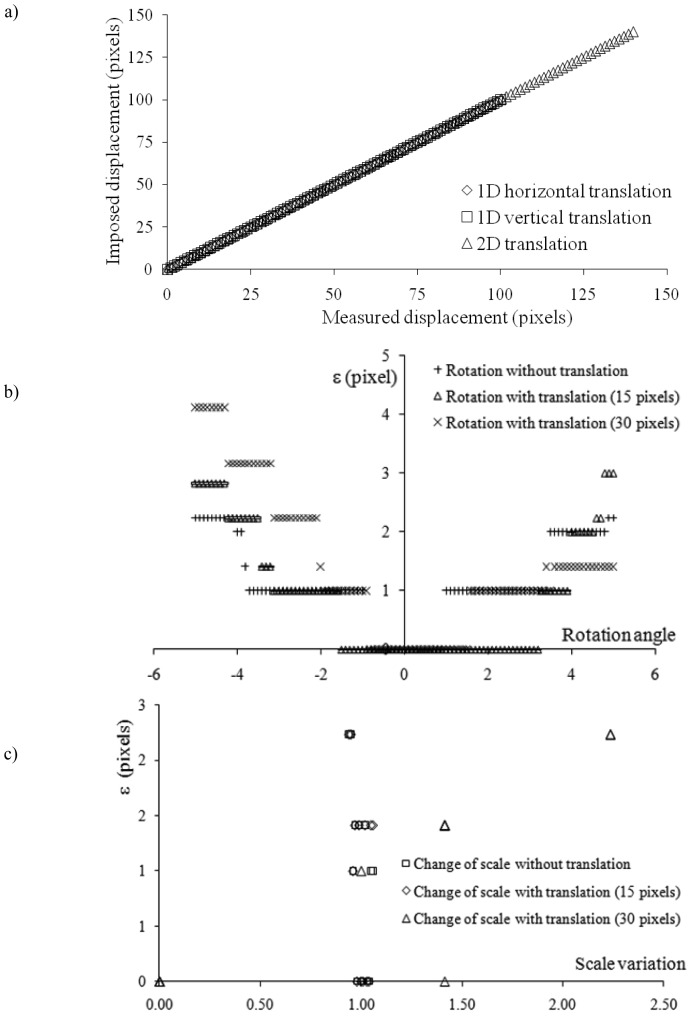
Comparison of imposed and measured displacements.

Figure 7 indicates the robustness of the method; *i.e.*, this figure can be used to determine the accuracy of the displacement inferred by the correlation function peak to the imposed one. The committed error is $\varepsilon =\sqrt{{\left({\text{d}}_{\text{x}}-{\text{i}}_{\text{x}}\right)}^{2}+{\left({\text{d}}_{\text{y}}-{\text{i}}_{\text{y}}\right)}^{2}}$ where the imposed displacement components are (d_x_,d_y_) and the displacement detected by the algorithm components are (i_x_,i_y_). It is worth recalling the uncertainty on the displacement measurement is of ±0.5 pixel. Figure 7(a) demonstrates that the proposed methodology works properly when images are shifted with respect to one another. For all translations in the x, y and oblique (45°) directions, the displacement inferred based on the correlation coefficient peak is equal to the imposed displacement. The bisector of the coordinate plane coincides with the three lines displayed in Figure 7(a) (the bisector is not shown to avoid confusion).

Figure 7(b,c) represent ε as a function of the rotation angle and scale variation, respectively. During the correlation of an image pair, one of which is rotated around its center, the algorithm detects a small displacement, even if no translation is imposed. The extent of the detected displacement increases when the rotation angle approaches the limits of the variation interval. For example, the detected displacement is small for rotations within the interval of −0.9° to 0.9°. Conversely, when the images are rotated and shifted, the detected displacement coincides with the imposed displacement within a larger rotation interval. Two translations, 15 pixels and 30 pixels, have been considered. In both cases, the second image was rotated within the interval of −5° to 5° with a step of 0.1°. In the former case (translation of 15 pixels), the displacement corresponding to the maximum correlation coefficient value is equivalent to the displacement imposed for rotations within the interval of −0.7° to 2.4°; in the latter case (translation of 30 pixels), the interval is slightly larger (−0.8° to 3.3°). The superposition of translation and rotation increases the consistency of the correlated images. Analogous conclusions can be drawn in the case of changes of scale in the second image with respect to the first one. The displacement corresponding to the maximum correlation coefficient ranges from zero (in case of no change of scale) to 2 pixels and 1 pixel in the x and y directions, respectively, reaching the lower extreme of the variation interval. Remarkably, the detected displacements are smaller if the second image is magnified with respect to its original size. The superposition of a translation across the images increases the consistency. Finally, we have investigated the ability of the algorithm to detect the rotation angle and the variation of scale applied to the original image; we also studied the capacity to provide the proper displacement in both the x and y directions.

**Figure 8.**
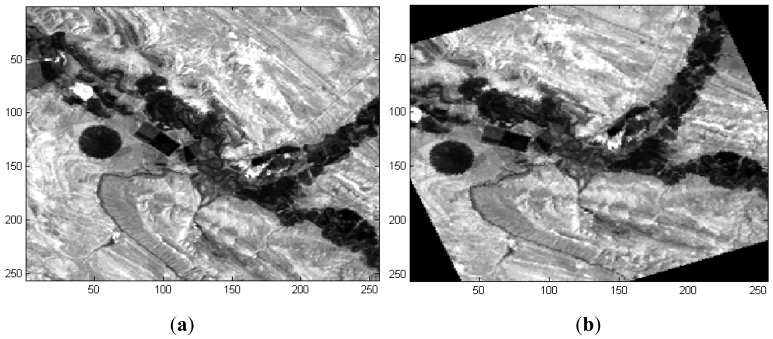
(**a**) First image of the pair; (**b**) second image of the pair rotated 21° counterclockwise, scaled 1.02 times the size of the original image and shifted 20 pixels in both the x and the y directions.

The image pair is shown in Figure 8. The second image of the pair is shifted 20 pixels in both the x and the y directions and is subsequently rotated 21° counterclockwise and scaled to 1.02 times the size of the original image. The rotation angle, which is largely outside the interval employed for the other tests, was chosen to emphasize the differences between the images.

**Figure 9.**
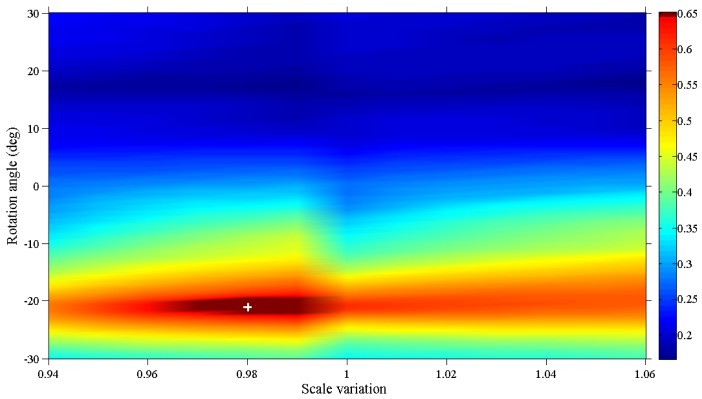
Map of the correlation coefficient maxima obtained via FFT of image pairs that are rotated and scaled by the quantities reported on the plot axes. The white cross identifies the rotation angle and scale variation, which provides the maximum correlation function value.

Figure 9 reports the color map of the correlation coefficient maxima detected via 2-DFFT. 2-DFFT is applied to the pair of images, the first of which is consistently the same as the image reported in Figure 8(a), whereas the second image is obtained by rotating and scaling the image shown in Figure 8(b) by the values reported on the axes of Figure 9.

**Figure 10.**
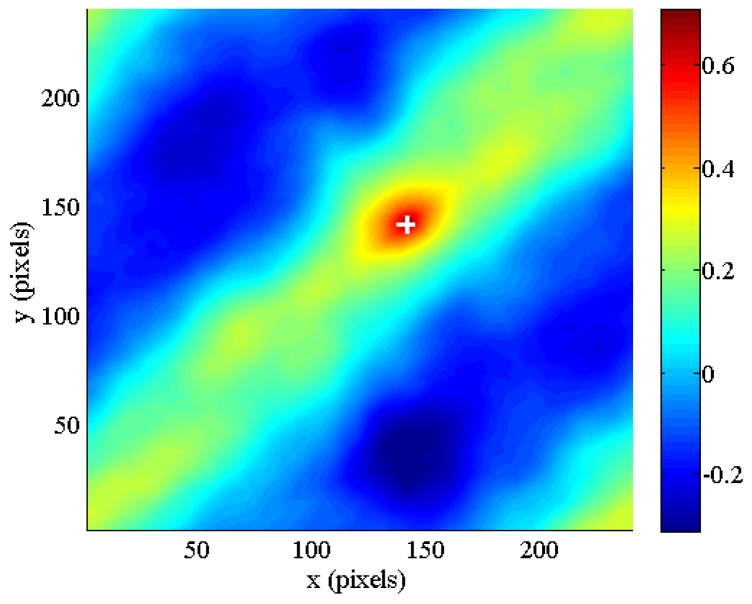
Cross-correlation map corresponding to a rotation angle of −21° clockwise and a scale of 0.98 times the size of the original image. The image is 240 × 240 pixels instead of 256 × 256 pixels, due to the change of scale.

The largest value in this map is highlighted with a white cross. The mosaicing algorithm is designed to select the rotation angle and the scale variation that provide the maximum value of the correlation function. In this case, the maximum corresponds to a rotation angle of −21° clockwise and a scale 0.98 times the size of the original image. As expected, this peak is shifted by 20 pixels in both the x and y directions (Figure 10).

## 5. Application to Real Data

The results of the ‘proximal sensing’ measurement campaign in San Teodoro (Olbia-Tempio—Sardinia) at a medium scale through the use of the spectrometer platform mounted on ultra-light aircraft are presented here. The area under investigation is almost flat. The objective of the measurement campaign is to categorize automatically all pixels in an hyperspectral cube into themes. The spectral pattern (*i.e.*, the set of radiance measurements obtained in the various wavelength bands for each pixel) is used as the numerical basis for categorization.

We have employed a supervised classification methodology. Supervised classification is comprised of two steps. The image analyst “supervises” the pixel categorization process by specifying various land cover types present in the scene. To perform this supervision, representative sample sites of known cover type, known as training areas, are used to describe the spectral attributes for each feature type of interest. Next, each pixel in the data set is numerically compared to each category that is identified and labeled with the name of the category with the greatest similarity [1].

**Figure 11.**
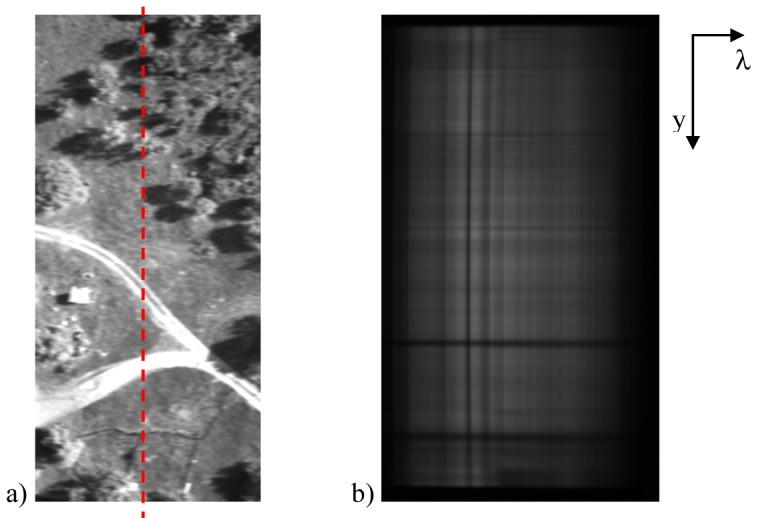
Sample images acquired by (**a**) the 4M60 camera equipped with a standard lens and (**b**) the VIS spectral imaging device.

The images used to build the mosaic show modest shading toward the periphery, which is caused by vignetting. This problem is modeled by a cos^4^(α) fall-off in intensity away from the principal point, assuming that the optic axis passes through the image center. No further image pre-processing was required. Figure 11(a) shows a sample image acquired by the 4M60 camera equipped with a standard lens and corrected for image noises. Figure 11(b) shows the corresponding image (along the dashed line in Figure 11(a)) acquired by the spectral imaging device for the visible range of the electromagnetic spectrum (400 nm to 1,000 nm). The size of imaged scene is determined by the width of the entrance aperture of the spectrograph and by the length of the slit [3]. NIR images from the field survey are unavailable, due to technical problems encountered during the measurement campaign. The spectral information is shown along the λ axis. Each column of the image is, therefore, representative of the reflectance characteristics at a given wavelength of the imaged portion passing through the spectrometer slit. Columns must be appropriately combined to reconstruct the image of the entire area at the given wavelength. This was accomplished by employing image mosaicing algorithm results.

**Figure 12.**
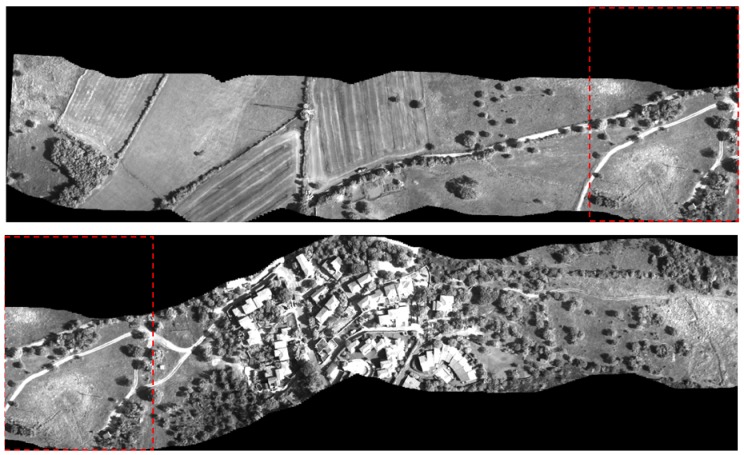
Result of the mosaicing procedure applied to 500 images acquired with the camera Dalsa 4M60 equipped with a standard lens. For a better interpretation of the image, the entire mosaic has been split into two parts; the common portion is highlighted within a dashed rectangular area.

The result of the application of the mosaicing code to a sequence of 500 images (extracted from the sequence of 1,500 images) acquired during the monitoring campaign is presented in Figure 12. For a better interpretation of the image, the entire mosaic has been split into two parts; the portion common to both images is highlighted within the dashed rectangular area. The use of a high spatial resolution acquisition sensor and a flight height of less than 500 m yields a ground resolution of less than 10 cm. The region represents an area recently affected by the construction of a residential area and a rural environment characterized by shrub vegetation mixed with large areas of lawn.

**Figure 13.**
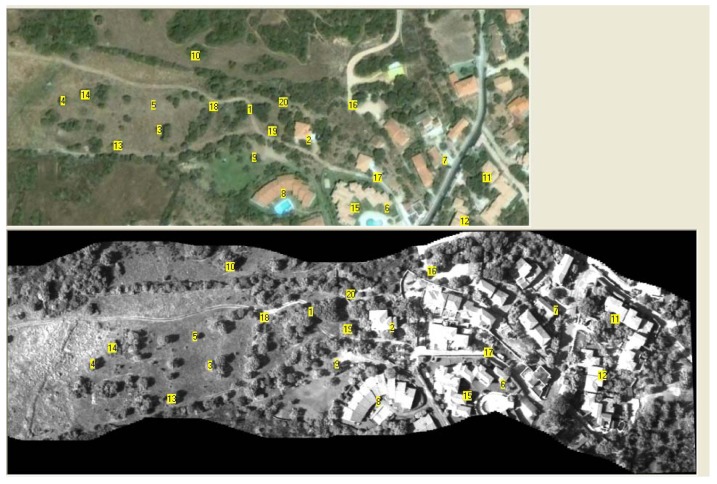
Map extracted from Google Earth employed for georeferencing both the mosaic and the images of the hyperspectral cube. The points employed for controlling the error in the georeferencing operation are shown in yellow.

**Figure 14.**
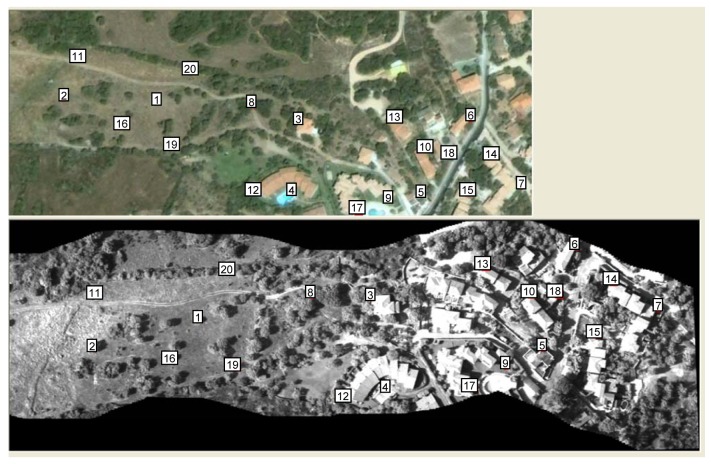
Map extracted from Google Earth employed for georeferencing both the mosaic and the images of the hyperspectral cube. The points employed for the geometric transformation are shown in white.

Figure 13 presents the map, extracted from Google Earth, which was employed for the georeferencing of both the mosaic and the images of the hyperspectral cube. For the sake of clarity, the procedure is described for only a portion of the mosaic presented above. The points employed for controlling the error in the georeferencing operation are shown in yellow. Figure 14 presents the points employed for the geometric transformation (in white).

The georeferencing procedure was applied four times using 7, 10, 15 and 20 points (Table 1). The mean deviation varies between 3.76 and 3.47 (3.61 on average) pixels. Given that one pixel in the Google map is equal to approximately 0.57 m, the deviation varies between 2.14 and 1.94 m. Changes due to an increase in the number of points employed for the georeferencing operation appear negligible, indicating that the distribution of the points is more important than the number of points.

Table 2 shows the results of the georeferencing operation when seven points distributed in the eastern (E) or western (W) portion of the map are used. The column labeled “C” reports previous results in which points were uniformly distributed within the area under investigation. In both cases, the results were significantly worse than when the test uses seven points that are well-distributed within the area. The increase in the error standard deviation from 3.76 to 12.87 (an increase of 242%) for points located in the eastern portion is significant. For points located in the western portion, the increase is smaller at 25.5%. It is worth noting that the deviation between calculated and control point coordinates occurs for many control points (3-4-7-11-13-16) independent of their position.

**Figure 15.**
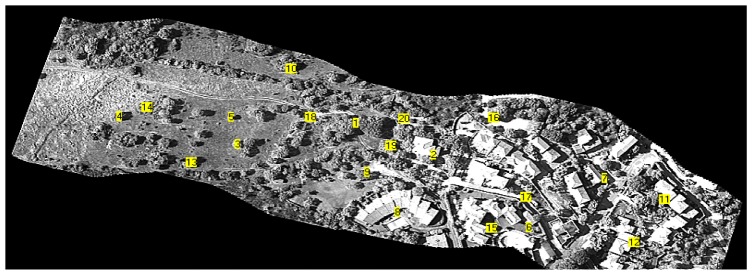
Georeferenced map.

Figure 15 presents the results of the georeferencing operation. As mentioned above, the geometrically corrected images may be transformed into a .kmz format to be overlapped with Google Earth maps.

**Figure 16.**
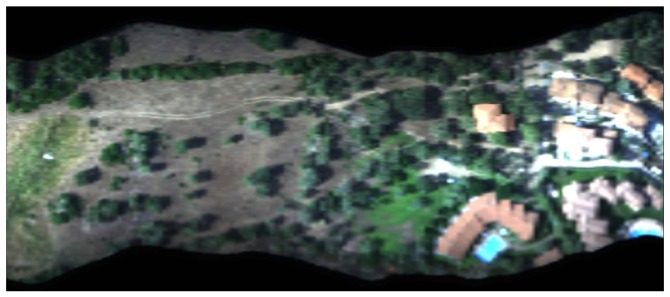
Hyperspectral cube representation in RGB using images at bands 660 nm (R), 560 nm (G) and 480 nm (B).

The information provided by the georeferincing procedure is subsequently employed to construct the hyperspectral cube. In Figure 16, the hyperspectral cube of the area under investigation (built with 61 images ranging from wavelengths of 400 nm to 1,000 nm with a step of 10 nm) is displayed in RGB using images at the 660 nm (R), 560 nm (G) and 480 nm (B) bands. To save space, the image was rotated.

**Figure 17.**
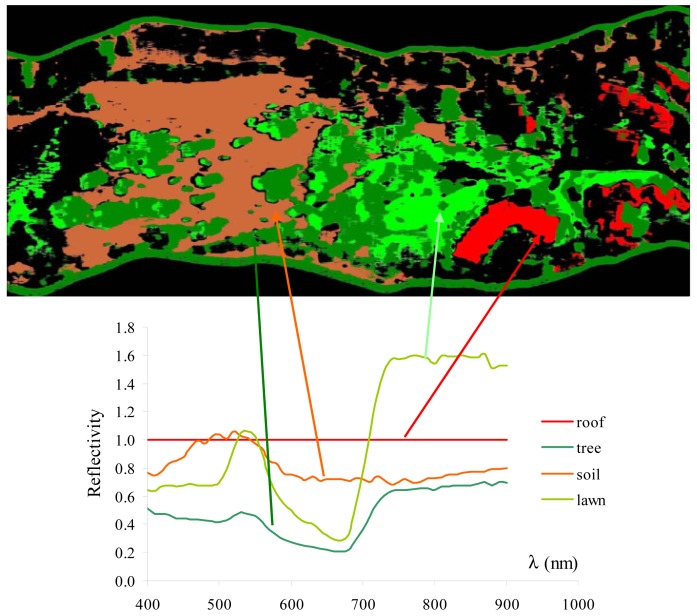
Map of the investigated area and spectral signatures.

To map the georeferenced area, the Maximum Likelihood classifier with four classes was employed. The classifier uses the distribution of data within each region of interest to calculate n-D probability functions for each class (where n represents the number of bands being used in the classification). Each pixel is assigned to the class for which the highest probability is calculated. It is common to have pixels unclassified with this method (black areas in the classification map). The result of this mapping and the spectral libraries for each class identified by the classification procedure are presented in Figure 17. The signatures were grouped on the same graph. The signature of the building roofs is flat and equal to one since it was employed for the radiometric calibration of the hyperspectral cube. The spectral libraries for vegetation show that the classifier is able to distinguish the lawns from shrubs. Those spectral signatures present the typical features of vegetation, *i.e.*, green peak, chlorophyll wells, red edge and NIR plateau. Their analysis allows the extraction of the indices that characterize the state of the vegetation. In conclusion, this classification is an excellent method for identifying the distribution of different types of framed ground surfaces.

## 6. Conclusions

The system developed in this study has several unique and qualifying characteristics: a lower cost compared to other systems available on the market; high spectral resolution; high spatial and temporal resolution; portability; and the system has been engineered such that it can be transported by ultra-light airplanes. The system is suitable for monitoring a range of small to large areas. The bandwidth is on the order of 10 nm (but it can be set to 3 nm), and the spatial resolution ranges up to the order of centimeters, allowing a great extent of detail in extracted information.

The mosaicing method employed in this study is designed for images that can be registered by means of a planar homography. The mosaic is obtained through a two-step procedure. First, the reciprocal position of subsequent images of the acquired sequence is determined with a 2-DFFT-based algorithm for detecting the maximum spatial correlation between image pairs. Second, the result of image mosaicing is georeferenced in the WGS-84 geographic coordinate system datum via the comparison to the reference map. The geometric registration with the reference image (a Google Earth map, in this case) is not influenced by the number of points employed; however, the distribution of the points has a large effect on the extent of error.

Possible improvements to the algorithm could address the first step of the image mosaicing. First of all, we are planning the implementation of sub-pixel displacement estimators to refine the displacement detection. Furthermore, the algorithm refinement will include an additional step after the mosaic is georeferenced to the WGS-84 datum. Each image of the acquired sequence will be compared to the corresponding reference map portion, and the consistency between the two compared images will be maximized. We expect that the reprojection of all views within the global frame will be associated with a smaller accumulation of error.

## Figures and Tables

**Table 1.** Deviation (in pixels) between points in the reference map and in the map resulting from the georeferencing operation.

**Control point**	**Number of points employed for georeferencing images**				**Mean deviation**

	**7**	**10**	**15**	**20**	
1	0.00	1.00	1.00	1.41	0.85
2	2.24	2.24	2.24	2.24	2.24
3	2.00	1.00	1.41	2.00	1.60
4	3.00	3.16	3.16	3.00	3.08
5	4.00	3.00	3.00	3.00	3.25
6	0.00	1.00	1.00	1.00	0.75
7	1.00	1.41	1.41	1.00	1.21
8	1.41	0.00	1.41	1.41	1.06
9	7.00	7.00	7.00	7.00	7.00
10	7.07	6.00	6.08	5.10	6.06
11	3.61	3.61	3.16	3.16	3.38
12	2.83	2.24	1.41	1.41	1.97
13	4.12	3.16	3.16	2.24	3.17
14	3.61	4.12	3.16	3.16	3.51
15	2.24	2.83	3.61	3.61	3.07
16	2.00	2.00	2.00	2.24	2.06
17	3.61	3.61	3.61	4.24	3.76
18	7.28	8.06	7.07	7.00	7.35
19	2.00	2.00	2.24	2.24	2.12
20	4.12	4.00	4.00	4.00	4.03
**Error standard deviation**	**3.76**	**3.67**	**3.54**	**3.47**	**3.61**

**Table 2.** Deviation (in pixels) between the calculated and control point coordinates.

**Control point**	**Deviation in pixels between the calculated and control point coordinates**		

	**W**	**E**	**C**
1	3.00	3.16	0.00
2	2.83	4.12	2.24
3	2.24	12.65	2.00
4	7.28	22.47	3.00
5	2.83	6.40	4.00
6	6.40	2.24	0.00
7	2.24	19.03	1.00
8	5.00	10.44	1.41
9	6.08	12.37	7.00
10	4.12	13.15	7.07
11	3.61	17.12	3.61
12	5.83	7.62	2.83
13	2.83	25.71	4.12
14	1.00	14.04	3.61
15	6.32	5.39	2.24
16	3.61	18.11	2.00
17	7.21	8.06	3.61
18	6.08	5.10	7.28
19	3.16	2.00	2.00
20	5.39	10.82	4.12
**Error standard deviation**	**4.72**	**12.87**	**3.76**
